# On the Origin of Iron/Sulfur Cluster Biosynthesis in Eukaryotes

**DOI:** 10.3389/fmicb.2019.02478

**Published:** 2019-11-08

**Authors:** Anastasios D. Tsaousis

**Affiliations:** Laboratory of Molecular and Evolutionary Parasitology, ResistAnce Pathogenicity and Infectious Diseases (RAPID) Group, School of Biosciences, University of Kent, Canterbury, United Kingdom

**Keywords:** last eukaryotic common ancestor, iron sulfur cluster biogenesis, sulfur mobilization machinery, iron sulfur cluster machinery, eukaryotic evolution, cytosolic iron/suphur cluster assembly machinery

## Abstract

Iron and sulfur are indispensable elements of every living cell, but on their own these elements are toxic and require dedicated machineries for the formation of iron/sulfur (Fe/S) clusters. In eukaryotes, proteins requiring Fe/S clusters (Fe/S proteins) are found in or associated with various organelles including the mitochondrion, endoplasmic reticulum, cytosol, and the nucleus. These proteins are involved in several pathways indispensable for the viability of each living cell including DNA maintenance, protein translation and metabolic pathways. Thus, the formation of Fe/S clusters and their delivery to these proteins has a fundamental role in the functions and the evolution of the eukaryotic cell. Currently, most eukaryotes harbor two (located in cytosol and mitochondrion) or three (located in plastid) machineries for the assembly of Fe/S clusters, but certain anaerobic microbial eukaryotes contain sulfur mobilization (SUF) machineries that were previously thought to be present only in archaeal linages. These machineries could not only stipulate which pathway was present in the last eukaryotic common ancestor (LECA), but they could also provide clues regarding presence of an Fe/S cluster machinery in the proto-eukaryote and evolution of Fe/S cluster assembly machineries in all eukaryotes.

## Introduction

Iron/sulfur (Fe/S) clusters are fundamental and ubiquitous factors. All living cells have biosynthetic machineries responsible for their assembly and delivery, since the individual components [iron (Fe) and sulfur (S)] are toxic for the cells themselves (Lill et al., 1999; Lill, 2009). Importantly, Fe/S clusters are essential factors of proteins involved in essential functions of the cell including, but not restricted to, photosynthesis, respiration, DNA replication and repair, and regulation of gene expression (Lill et al., 2012). Eukaryotes are not the exception to this paradigm. The typical Fe/S biosynthetic machineries found in bacteria and archaea have also been identified in eukaryotes, but compartmentalization and evolution of these machineries in several eukaryotes are still under investigation. A typical eukaryotic cell harbors the iron-sulfur cluster (ISC) in the mitochondria and the cytosolic iron/suphur cluster assembly (CIA) machinery in the cytosol, while plastid-carrying cells also harbor the sulfur mobilization (SUF) machinery in their plastids.

Among those, the ISC machinery has been considered to be the reason for the existence of mitochondria (Lill et al., 1999; Lill, 2009; Hjort et al., 2010) and fundamental for the evolution of eukaryotes. Nonetheless, what happens when a eukaryote does not harbor any mitochondria (Karnkowska et al., 2016)? Could this organism provide some clues about the presence of Fe/S biosynthetic machineries in the early eukaryotes and their role in the evolution of the eukaryotic cell?

## Iron-Sulfur Cluster Assembly in Mitochondrial Diversity

It is widely accepted that mitochondria originated from or within the alpha-proteobacteria (Gray et al., 1999, 2001; Gawryluk, 2018; Martijn et al., 2018), whereby the latter was “engulfed” by a eukaryotic host and potentially gave rise to the last eukaryotic common ancestor (LECA). Nevertheless, questions regarding why, how, and when this event took place are still under debate (Gray et al., 2001; Embley and Martin, 2006; Lane and Martin, 2015, 2016; Martin et al., 2016; Pittis and Gabaldon, 2016; Gabaldon, 2018). It is quite apparent from accumulated data that the acquisition of mitochondria has been the decisive step in eukaryogenesis (Martin et al., 2016). One hypothesis postulates that the mitochondria fulfilled energy requirements of the cell thus their presence provided a selective advantage to the organisms bearing them to become eukaryotes (Lane and Martin, 2015, 2016; Pittis and Gabaldon, 2016). Another hypothesis, which does not exclude others, suggests that the reason for the existence of mitochondria could have been the assembly of Fe/S clusters (Lill et al., 1999), the latter being the only mitochondrial biosynthetic pathway that is essential for survival of eukaryotic cells. So far, this has been shown experimentally in yeast (Braymer and Lill, 2017), mammalian cells (Rouault and Maio, 2017), and trypanosomes (Pena-Diaz and Lukes, 2018).

Further support to this hypothesis arose from investigations in previously considered “primitive” amitochondriate eukaryotes. These organisms were shown to harbor mitochondrial-related organelles (MROs), a secondarily reduced form of mitochondria, including hydrogen producing organelles called hydrogenosomes in *Trichomonas* (Muller, 1973), or highly reduced remnant organelles called mitosomes, which were found in *Giardia* (Tovar et al., 2003); *Microsporidia* (Williams et al., 2002; Tsaousis et al., 2008), and *Entamoeba* (Tovar et al., 1999). Whether a “primitive” amitochondriate eukaryote could exist or not is still under debate (Margulis et al., 2006). Nonetheless, a eukaryote that secondarily lost its mitochondria was identified recently (Karnkowska et al., 2016). Interestingly, the only biosynthetic pathway conserved in all these organelles is the assembly of Fe/S clusters, providing further support on the necessity/importance of this machinery for cell viability. From an evolutionary standpoint, it will be important to elucidate how the eukaryotic cell supported its needs for Fe/S clusters, before the acquisition of mitochondria. To provide insight on this matter, I will first need to examine the distribution of various Fe/S cluster machineries in eukaryotic cells and their necessity to the host’s functions, followed by various theories on the evolution of Fe-S cluster machineries across eukaryotes.

## Mitochondrial Iron/Sulfur Cluster Machinery

**Figure 1:**
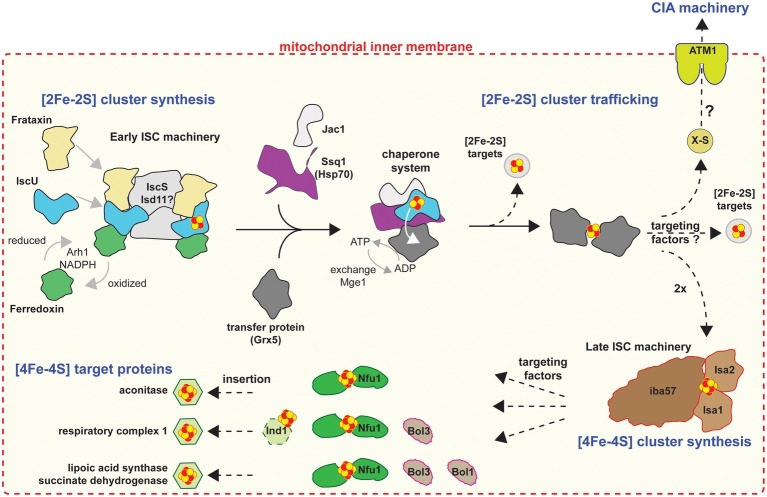
Cartoon model of the mitochondrial Fe/S protein assembly process. Figure was produced based on Braymer and Lill (2017). A cascade of ISC proteins is required for the *de novo* synthesis of [2Fe-2S] and [4Fe-4S] clusters and their proper trafficking to target apoproteins in mitochondria. Initially, a [2Fe-2S] cluster is synthesized by the early ISC machinery, composed of the Isu1 scaffold protein requiring sulfide from the cysteine desulfurase complex Nfs1-Isd11-Acp1, electrons from the transfer chain NADPH-Arh1 and the ferredoxin Yah1, and the regulator and/or iron donor Yfh1. The Isu1-bound [2Fe-2S] cluster is then delivered to the monothiol glutaredoxin Grx5, a reaction accomplished by the Hsp70 chaperone Ssq1 with the help of the J-type co-chaperone Jac1. This reaction is dependent on ATP hydrolysis by Ssq1. The exchange factor Mge1 facilitates the exchange of ADP for ATP. The resulting bridging [2Fe-2S] cluster on a Grx5 dimer is inserted directly into [2Fe-2S] recipient apoproteins or trafficked to the late ISC machinery for [4Fe-4S] cluster biogenesis. The early ISC machinery, including the chaperones and Grx5, is also responsible for generating the component X-S for transport of sulfur out of the mitochondria to the CIA machinery for cytosolic-nuclear Fe/S protein biogenesis. The late ISC machinery consists of the yet structurally and functionally uncharacterized Isa1-Isa2-Iba57 complex and is needed for the generation of [4Fe-4S] clusters. Trafficking and insertion of the [4Fe-4S] clusters into target Fe/S proteins are facilitated by specific ISC targeting factors, such as Nfu1, the complex I-specific Ind1, and the Bol proteins. Dashed arrows indicate steps that remain poorly elucidated on the biochemical level.

All mitochondria investigated so far possess some semblance of an Fe/S cluster biosynthetic pathway for *de novo* assembly of Fe/S clusters into organellar apo-proteins (see below) but potentially for the support of cytosolic and nuclear apo-proteins as well (Lill, 2009; Ali and Nozaki, 2013). The typical mitochondrial machinery is the iron-sulfur cluster (ISC), which is comprised of 18 (currently known in yeast) proteins (Braymer and Lill, 2017), all of which are involved in the biogenesis and trafficking of clusters in mitochondria (Figure 1). The process is divided into four stages (for detailed review, see Braymer and Lill, 2017): (1) *de novo* [2Fe-2S] cluster synthesis; (2) trafficking of [2Fe-2S] clusters and insertion into mitochondrial apo-proteins, or mitochondrial export of an as yet unknown sulfur-containing species (X-S) to the cytosol; (3) conversion of [2Fe-2S] into [4Fe-4S] clusters; and lastly (4) trafficking of [4Fe-4S] clusters and insertion into mitochondrial [4Fe-4S] apo-proteins (e.g., lipoate synthase, succinate dehydrogenase, and components of respiratory complex I). Most organisms harboring mitochondria encode some of these components, including organisms with remnant mitochondria such as *Giardia* (Tovar et al., 2003), *Cryptosporidium* (Miller et al., 2018), and *Microsporidia* (Goldberg et al., 2008; Freibert et al., 2017), in which ISC stages 3 and 4 are lacking ([4Fe-4S] cluster synthesis and targeting; Figure 1), due to the lack of mitochondrial apo-proteins requiring [4Fe-4S] clusters.

## Cytosolic Iron/Sulfur Cluster Machinery

**Figure 2:**
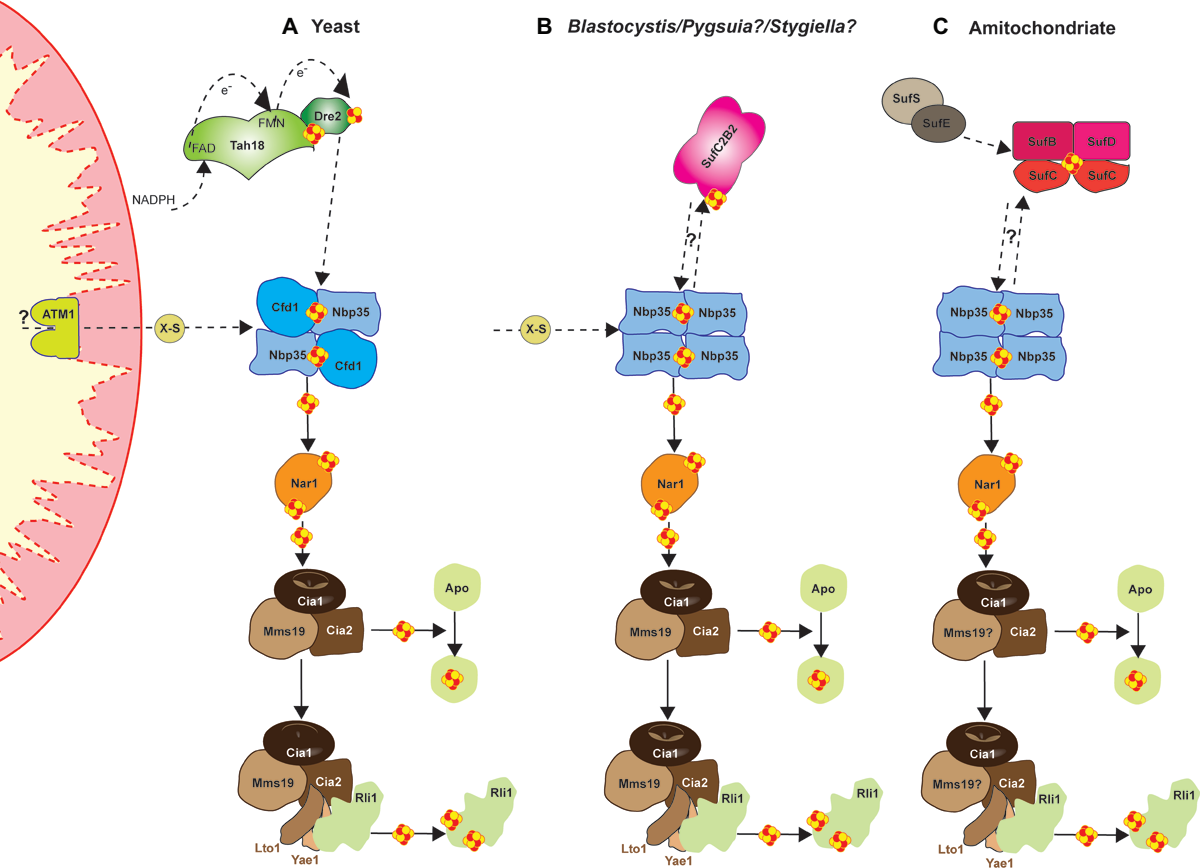
Cartoon demonstrating the current model, based on Braymer and Lill (2017), for the mechanism of yeast cytosolic-nuclear Fe-S protein biogenesis **(A)** and a hypothetical model for the *Blastocystis* **(B)**, and the amitochondriate *Monocercomonoides* **(C)**. Assembly of extra-mitochondrial Fe-S proteins is catalyzed by the cytosolic iron-sulfur protein assembly (CIA) machinery in an ISC-dependent manner. Several CIA protein complexes support different stages of the process. Initially, a bridging [4Fe-4S] cluster is assembled on the Cfd1-Nbp35 scaffold complex, but the bridging cluster binds only transiently. Nbp35 contains another stably bound [4Fe-4S] cluster at its N-terminus. Cluster assembly on Cfd1-Nbp35 depends on the molecule X-S from the mitochondrial ISC machinery. Further, the electron transfer chain from NADPH *via* the diflavin reductase Tah18 and the Fe-S protein Dre2 is needed. In a second step, the transiently bound [4Fe-4S] cluster of Cfd1-Nbp35 is transferred to and inserted into apoproteins by the Fe-S protein Nar1, and the CIA targeting complex consisting of Cia1, Cia2, and Mms19. Maturation of the essential Fe-S protein Rli1 additionally depends on the function of the two specific adaptor proteins Yae1 and Lto1. The Yae1-Lto1 complex uses a unique binding cascade to recruit Rli1 to the CIA targeting complex for Fe-S cluster insertion.

All eukaryotes require a cytosolic Fe/S cluster (CIA) machinery to support cytosolic and nuclear Fe/S cluster proteins (Tsaousis et al., 2014). So far, 11 proteins have been identified in both mammals and yeast as responsible for synthesis, trafficking, and insertion of clusters in the cytosol and the nucleus (Braymer and Lill, 2017; Tonini et al., 2018). Of these, several CIA protein complexes support different stages in the process (Figure 2A). For example, a bridging [4Fe-4S] cluster is assembled on the Cfd1-Nbp35 complex, which depends on the as yet unidentified molecule X-S from the mitochondrial ISC machineries. Subsequently, the electron transfer chain from NADPH *via* the diflavin reductase Tah18 and the Fe/S protein Dre2 is required. In the next phase, the transiently bound [4Fe-4S] cluster of Cfd1-Nbp35 is transferred to and inserted into apo-proteins by the Fe/S protein Nar1, and the CIA targeting complex consisting of Cia1, Cia2, and Mms19 (Stehling et al., 2012, 2013). This entity also binds the Lto1-Yae1 adapter complex *via* a conserved C-terminal tryptophan in Lto1 to recruit the ABC protein Rli1 (participates in ribosome assembly and ribosome recycling) for dedicated assembly of its two [4Fe-4S] clusters (Lill et al., 2015; Paul et al., 2015). The CIA machinery may also support ATP-dependent DNA helicases such as Rad3, XPD, FANCJ, and RTEL1, which are involved in DNA damage repair and telomere maintenance (Rudolf et al., 2006). Interestingly, mitochondria or related organelles, such as hydrogenosomes and mitosomes (see above) seem to be essential for the support of the CIA machinery in the biogenesis of cytosolic and nuclear Fe/S clusters (Stehling et al., 2014; Tsaousis et al., 2014; Freibert et al., 2017). Despite this, organisms harboring these “reduced” mitochondria appear to lack certain components of the CIA machinery (e.g., Tah18, Dre2, and Cfd1) that are otherwise essential in mammals and yeast (Tsaousis et al., 2014; Vacek et al., 2018). Even more intriguingly, microbial organisms such as cryptophytes and chlorarachniophytes that harbor cytosols from two organisms (main and cytosol of their phototrophic symbiont), seem to have two diverse and functional CIA machineries—one in each compartment—which are supported by their corresponding organelles (Grosche et al., 2018).

## Plastid Iron/Sulfur Cluster Machinery

**Figure 3:**
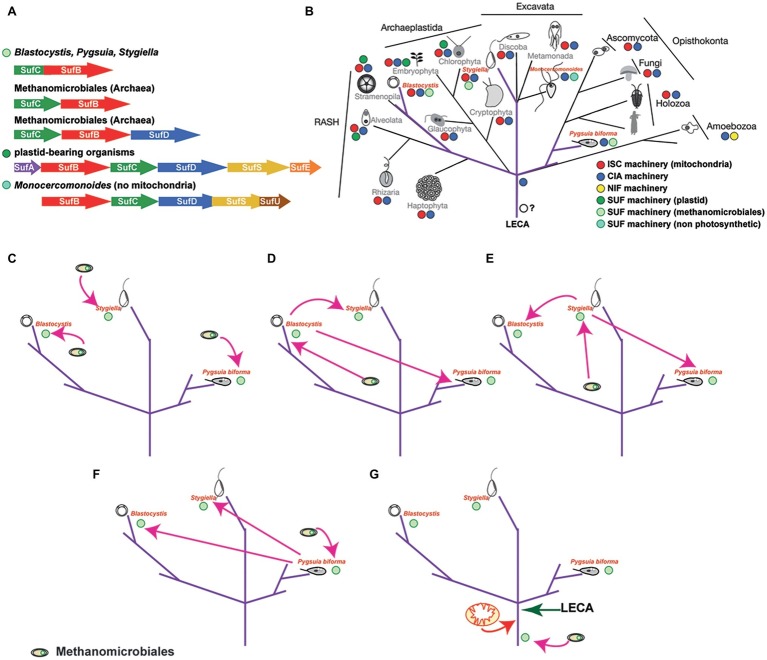
The distribution of the SUF system amongst microbes and scenarios on the evolution of the SUF machinery in eukaryotes. **(A)** The distribution of the SUF system amongst microbial genomes (based on Tokumoto et al., 2004). Since the sufBC-like genes are found in all species encoding this system, it has been speculated that these genes were components of the primitive system, which was further evolved through the recruitment of other components such as SufA, SufE, and SufS (e.g. *E. coli* Suf system). The fused genes found in *Blastocystis, Pygsuia*, and *Stygiella* genomes/transcriptoms corresponding to the SufCB operon in *Methanomicrobiales*. The SufCB operonencodes two out of the six proteins of the SUF system (e.g *E. coli* or plastid bearing organisms) and is part of the Suf system found in extremophiles. **(B)** The eukaryotic tree of life demonstrating the distribution of the various Fe/S cluster biosynthetic pathways in eukaryotes, highlighting (purple color) the unique distribution of the SUF system across eukaryotes. Relationships between eukaryotes are based on recent concatenated phylogenetic results (Burki et al., 2016). **(C)** This scenario suggests that the common ancestor of *Blastocystis* has acquired the fused gene from a methanoarchaeon, while *Pygusia* and *Stygiella* independently acquiring the SufCB fused gene from an organism from the same group of *Methanomicrobiales* as well. **(D)** In this scenario, the last common ancestor of *Blastocystis* acquired the SufCB fused gene from an organism from the group of *Methanomicrobiales* which was laterally gene transferred to *Pygsuia* and *Stygiella*. **(E)** In this scenario, *Stygiella* acquired the SufCB fused gene from an organism from the group of *Methanomicrobiales* which was laterally gene transferred to *Pygsuia* and the last common ancestor of *Blastocystis*. **(F)** In this scenario *Pygsuia* acquired the SufCB fused gene from an organism from the group of *Methanomicrobiales* which was laterally gene transferred to *Stygiella* and the last common ancestor of *Blastocystis*. **(G)** In this scenario, the methanoarchaeal SufCB was either present in the last eukaryotic common ancestor or was acquired later before the split of the various eukaryotic lineages.

Apo-proteins in plastids and plastid-related organelles are supported by the sulfur mobilization (SUF) machinery, which was acquired from *Cyanobacteria*. The six major proteins that encompass the bacterial-type SUF machinery are also present in plastids (SufA, SufB, SufC, SufD, SufE, and SufS; Figure 3A), one of which (SufC) is commonly encoded by the plastid genome (Le Corguille et al., 2009). Using genetic and biochemical investigations in prokaryotes, it was shown that SufE and SufS are involved in the sulfur mobilization from cysteine, while SufB, SufC, and SufD form a complex where SufB harbors both the *de novo* assembled Fe/S clusters and a flavin redox cofactor (Couturier et al., 2013). However, recent experimental structural studies have shown a dynamic motion of the SufB_1_-SufC_2_-SufD_1_ complex that could be universally applicable to all the SUF systems, including the archaeal SufB_2_-SufC_2_ complex (Hirabayashi et al., 2015) (discussed below). In addition, SufA could act as a carrier protein, along with numerous other carrier proteins that are currently found (Fontecave et al., 2005; Wollers et al., 2010; for review, see Couturier et al., 2013). As such, the plastidial Fe/S assembly machinery has been mostly characterized in *Arabidopsis thaliana*, where 15 proteins have been experimentally localized and one of which (SufSE) was shown to be targeted in both the plastids and mitochondria (Balk and Pilon, 2011; Couturier et al., 2013). To that end, the plastidial Fe/S cluster assembly is responsible for the support of housekeeping apo-proteins of the organelle and currently is unclear if it can support the CIA machinery in cytosol of the cells (similar to the ISC machinery).

## Iron/Sulfur Cluster Assembly in Amitochondriates

The discovery of a eukaryote that secondarily lost its mitochondria (Karnkowska et al., 2016) raises the question of Fe/S cluster biosynthesis in this organism, since this is the only biosynthetic function found in all mitochondria-related organelles investigated so far (Hjort et al., 2010; Santos et al., 2018). The oxymonad *Monocercomonoides* sp. (currently named *M. exilis*; Treitli et al., 2018) is the first eukaryotic organism with no microscopic evidence for the existence of a mitochondrion. This finding was further supported by extensive genome surveys that failed to find any mitochondrial proteins, including homologues of the mitochondrial ISC pathway (Karnkowska et al., 2016). Despite this, the genome of *Monocercomonoides* does encode components of the CIA machinery (Figure 2C), in addition to homologs of a SUF system (Figures 3A,B). The origin of these SUF homologues though unclear seems to be bacterial (Karnkowska et al., 2016) (see below). Due to the lack of an *in situ* transfection system, *Monocercomonoides* SufC and SufB homologs were heterologously expressed in *Trichomonas vaginalis* and *Saccharomyces cerevisiae*, whereby they both localized in the cytosol of both organisms (Karnkowska et al., 2016).

Recent investigations by Vacek et al. (2018) demonstrated that oxymonads and organisms (Preaxostyla group, *Metamonada*, and *Excavata*) related to *M. exilis* also harbor an SUF machinery (Vacek et al., 2018). Genomic and transcriptomic surveys have shown the presence of components of the SUF machinery in six additional closely related species, suggesting that transition from ISC to SUF preceded the last common ancestor of the lineage (Vacek et al., 2018). A follow-up inventory of all the homologues of the CIA machinery in these organisms showed that its major components are still present, consistent with previous observations that the lack of mitochondria or more specifically of the ISC machinery did not have any effect in the maturation of cytosolic Fe/S proteins (Vacek et al., 2018).

## Exceptions to the *Status Quo* (Alternative Directions)

### The Case of *Entamoeba* and *Mastigamoeba*

In addition to the machineries described above, some organisms have acquired new processes for the *de novo* assembly of their Fe/S clusters. The genomes of the amoebozoans *Entamoeba histolytica* and *Mastigamoeba balamuthi* (both thriving in low-oxygen environments) do not encode any components of the ISC machinery and instead they harbor a nitrogen fixation (NIF) machinery that was laterally acquired from an epsilon proteobacterion (Ali et al., 2004; van der Giezen et al., 2004). Components of the machinery were shown to localize in the mitosome of *E. histolytica* (Maralikova et al., 2010) (though this is still under debate; Nyvltova et al., 2013), while replica components of *M. balamuthi* were shown to localize in both the cytosol and its hydrogenosomal-like structures (Nyvltova et al., 2013). It is still unclear whether the function of a NIF system could be more advantageous over the ISC system, but it seems to be the “preferred” way in this lineage. Despite this alteration, components of the CIA machinery are present in both organisms (Tsaousis et al., 2014; Pyrih et al., 2016) (with the exception of Tah18, Dre2, and Cfd1), suggesting that ISC machinery might not (as previously thought; Lill et al., 1999) be indispensable for the function of the CIA machinery.

### The Case of *Blastocystis*, *Pygsuia*, *Stygiella*, and Others?

*Blastocystis* is an obligatory anaerobic stramenopile. *Blastocystis* was the first non-photosynthetic eukaryotic organism to be shown to encode an ancient SUF system (Tsaousis et al., 2012), in addition to an ISC machinery that is localized in mitochondria (Tsaousis et al., 2012) and a CIA machinery that is localized in the cytosol (Tsaousis et al., 2014). The SUF system of *Blastocystis* is similar to the one of *Methanomicrobiales* in that both display fusion of the *SufC* and *SufB* genes. Phylogenetic analysis showed that both *Blastocystis* homologues grouped with those of the archaea into a strongly supported clade, indicating lateral acquisition of the gene from *Methanomicrobiales* (Tsaousis et al., 2012). The fused gene is found in the genomes of all *Blastocystis* subtypes, in addition to the genome of *Proteromonas lacertae* (found in BioProject: PRJNA386230), a Stramenopile species closely related to *Blastocystis*. Functional characterization of the *Blastocystis* protein showed that it binds [4Fe-4S] clusters and has ATPase activity. The protein was shown to localize in the cytosol of the parasite and to be overexpressed under oxygen-stressed conditions (Tsaousis et al., 2012). This was unsurprising, since in various bacteria, it has been demonstrated that the machinery is overexpressed under oxygen stress or iron depletion conditions, in order to support the potentially damaged apo-proteins of the cell (Rangachari et al., 2002; Mettert et al., 2008).

Following its discovery in *Blastocystis*, a fused *SufCB* gene was later found in other distantly related microbial eukaryotes. The first was the breviate *Pygsuia biforma*, a free-living anaerobe, but aerotolerant amoeboid flagellate isolated from hypoxic marine sediments. The organism branches at the base of the eukaryotic supergroup Obazoa, which is comprised of animals, fungi, and apusomonads (Figure 3B). The *P. biforma* genome encodes two homologues of the protein (Stairs et al., 2014). Localization experiments showed that one homologue localizes in mitochondria, while the other localizes in the cytosol (Stairs et al., 2014). Phylogenetic analysis showed that both *P. biforma* homologues branch closely with those of *Blastocystis*. Interestingly, analysis of the RNA-seq data did not show expression of any of the components of the mitochondrial ISC machinery, while components of the CIA machinery (Cia1, Nbp35, Cfd1, Nar1, Cia2, and Met18) were present (Stairs et al., 2014).

A fused SufCB gene was also found in *Stygiella incarcerata* along with genes encoding components of the mitochondrial ISC machinery (Leger et al., 2016). *Stygiella incarcerata* a microaerophilic jakobid flagellate inhabiting anoxic environments and is distantly related to Stramenopiles and Breviata (e.g., *Blastocystis* and *Pygsuia*, respectively; Figure 3B). The SUFCB gene of *S. incarcerata* displayed the same characteristics as the homologues of *Blastocystis* and *Pygsuia*, and it lacked mitochondrial targeting peptides suggesting a potential cytosolic localization. While the authors did not find any introns in the transcriptome derived fused gene, data from the closely related jakobid *Velundella trypanoides* (found in BioProject: PRJNA268717) also demonstrated the presence of a homologue (Leger et al., 2016), suggesting that the gene is likely not a contaminant. Phylogenetic analysis showed that the SUF eukaryotic homologues from *Blastocystis*, *Pygsuia*, and *Stygiella* formed a strongly supported clade, with *Methanomicrobiales* as a well-supported sister group (Leger et al., 2016), consistent with previous observations (Tsaousis et al., 2012; Stairs et al., 2014). How is it possible for organisms that are so distantly related to have a SUFCB homologue?

Various scenarios could explain the presence of this machinery in at least three eukaryotic lineages. Herein, I will discuss three scenarios (Figures 3C–G) while providing pros and cons for each hypothesis:

#### First Theory

All three organisms (or their ancestors) acquired the methanoarchaeal SufCB independently, likely while inhabiting the same environmental niche (Figure 3C). This scenario suggests three independent transfers: once in the common ancestor of *Blastocystis* and *Proteromonas*, once in *Stygiella*, and once in *Pygsuia*. Each transfer would require co-existence of the donor lineage with each eukaryote separately. Consequently, this setting implies that the ancestors of these organisms co-habituated in similar environments with *Methanomicrobiales*, which allowed for transfer and incorporation of genes in their genomes. The intriguing question, under this scenario, is why only a single fused gene was transferred or incorporated from these methanomicrobes in the genomes of diverse protozoa lineages (Tsaousis et al., 2012)?

#### Second Theory

The methanoarchaeal SufCB gene was acquired by one of the three eukaryotic organisms (or their ancestors) and then laterally transferred to the others (Figures 3D–F). It is well established that lateral gene transfer events from eukaryotes to eukaryotes are not as uncommon as it was once thought (Danchin, 2016; Eme et al., 2017; Leger et al., 2018). This type of scenario requires that at least two of the protists co-habited with the donor lineage in the same or similar niches at some point of their life cycles. For example, *Blastocystis* and *Proteromonas* spend the majority of their life cycle in the gut of various organisms. Nonetheless, *Blastocystis* is excreted in the environment as a cyst. If cysts were shed in hypoxic environments, then the possibility of *Pygsuia* and *Stygiella* encountering *Blastocystis* (or its ancestor) and subsequently exchanging genetic material is not entirely far-fetched. Interestingly, with the exception of the SufCB gene, to our knowledge, no other genes share the same origins (or clustering) in these three groups.

#### Third Theory

The methanoarchaeal SufCB was present in the last eukaryotic common ancestor (LECA) (Figure 3G). The LECA had to have a machinery for the assembly of Fe-S clusters to support its apo-proteins, even before the acquisition of the alpha-proteobacterium that gave rise to the present-day mitochondria. Notably, it has been suggested that the CIA machinery, which is present in all eukaryotes investigated so far is a eukaryotic innovation (Tsaousis et al., 2014; Freibert et al., 2017). Since the ISC machinery is found only in mitochondria and the NIF machinery is only present in two closely related organisms, it is unlikely that either one was present in LECA. Thus, an ancestral SUF machinery, which is commonly found in archaea (Outten, 2015), could have been present in LECA. Considering that *SufCB* is not only the most “ancient machinery” (Tokumoto et al., 2004) among all biosynthetic apparatuses, but also the most widespread across lineages, it is plausible that the SufCB was present in the common ancestor of eukaryotes as well. The machinery could have either been acquired by a methanoarchaeon or it could have been present in the archaeal group that gave rise to modern eukaryotes (Spang and Ettema, 2017; Zaremba-Niedzwiedzka et al., 2017; Eme et al., 2018). This scenario could explain the presence of a biosynthetic machinery in three distantly related eukaryotic lineages, but it also infers multiple losses of this machinery in the rest of the lineages. Under this scenario, the case of oxymonads is of interest (Karnkowska et al., 2016; Vacek et al., 2018). How can a separate origin of SUF be explained? One explanation would be that the ancestrally acquired SUF was lost and a SUF of different origin was acquired upon loss of mitochondria. Thus, I hypothesize that eukaryotes maintain the chassis that would allow reacquisition of SUF-like machinery. This hypothesis could be tested by incorporating the eukaryotic SUF machineries in various model organisms across the eukaryotic tree of life (e.g., *Saccharomyces*, *Trypanosoma, Tetrahymena*, and *Dictyostelium*). It is worth mentioning that the third theory does not necessary exclude the other theories above.

## Discussion: Iron/Sulfur Cluster Biosynthesis During the Evolutionary History of Eukaryotes

Given the discovery of this fused gene in diverse lineages of eukaryotes, speculative scenarios propose an initial transfer of the SufCB from an archaeal source into an ancestral microbial eukaryote (Figures 3C,G) and/or lateral gene transfer events to other eukaryotes (Tsaousis et al., 2014; Leger et al., 2016) (Figures 3D–F). Nevertheless, it is imperative to highlight the importance of this pathway in the evolution and adaptation of eukaryotes.

The last eukaryotic common ancestor (LECA) lived about 1.8 billion years ago (Betts et al., 2018) and seems to have been more complicated than was previously thought (Koonin, 2015). It has been speculated that LECA contained organelles and functions that even mirror some of the current microbial eukaryotes, based on comparative genomic analyses with the closest archaeal-relative lineage, the *Lokiarchaeota* (Spang et al., 2015, 2017, 2018; Zaremba-Niedzwiedzka et al., 2017; Eme et al., 2018; Eme and Ettema, 2018). Among those, it is currently suggested that LECA possessed mitochondria, endomembrane system along with nucleus, actin cytoskeleton, endocytosis and/or phagocytosis, and a ubiquitin network (Embley and Williams, 2015; Koonin, 2015; Spang et al., 2015; Akil and Robinson, 2018; Eme and Ettema, 2018). Metabolically, based on investigations in *Lokiarchaeota*, LECA could have been transitioning from anaerobic to aerobic metabolism (due to the acquisition of the mitochondria; aerobic respiration) with a potentially hydrogen-dependent autotrophic lifestyle (Martin et al., 2016; Sousa et al., 2016). Some of these pathways need enzymes (apo-proteins) that require Fe/S clusters in order to function, including DNA/RNA polymerases and anaerobic proteins (e.g., pyruvate ferredoxin oxidoreductase; PFO), which have been identified in *Lokiarchaeota* (Sousa et al., 2016). LECA must have harbored a biosynthetic pathway to support the assembly and trafficking of these Fe/S clusters. The presence of a SUF-like machinery in LECA is plausible, since it is the most common machinery amongst archaeal lineages and is also not compartmentalized in most eukaryotes (Tsaousis et al., 2012; Stairs et al., 2014; Karnkowska et al., 2016; Leger et al., 2016). Footprints of this ancient machinery still remain in modern eukaryotes and it is not an invalid prediction that more organisms having this machinery will be discovered. Whether the machineries that are present in *Blastocystis/Proteromonas*, *Pygsuia*, and *Stygiella* lineages are remnants of the initial machinery (LECA) or later acquisitions (see scenarios Figures 3C–G) will need further investigations; current data clearly illustrate that the CIA and SUF-like machineries can clearly co-exist (Tsaousis et al., 2012, 2014; Stairs et al., 2014; Karnkowska et al., 2016; Leger et al., 2016; Vacek et al., 2018).

It is also important to note that SUF-like machineries have been shown to be upregulated under oxygen stress conditions to support the potential degradation of Fe-S clusters of proteins (Rangachari et al., 2002; Mettert et al., 2008). This function/support would have been essential during the transformation of proto-eukaryotic cells to LECA, since during that period there would have been a transition to increasing concentrations of oxygen (Lane and Martin, 2016). A SUF-like machinery would have been able to compensate for the potential damage of Fe/S clusters from oxygen allowing cells to slowly adjust to their new environments. In parallel, acquisition of mitochondria provided not only an oxygen protective compartment for the formation of Fe/S clusters, but also the ISC machinery as well (Lill et al., 1999, 2015). Later on, adaptation of these cells to oxygen rich environments and expansion of the CIA machinery in the cytosol along with its ability to “communicate” with the mitochondrial ISC machinery (e.g., ATM1 for transfer of X-factor; Figures 1, 2), resulted into the SUF-like machinery becoming redundant to the ancestors of most eukaryotic lineages. Eukaryotes that still remained under oxygen depleted conditions either retained the SUF-like machinery (scenario Figure 3G) or later acquired a homologue of this (Vacek et al., 2018).

Here, I propose various scenarios on the evolution of the Fe-S cluster machineries in eukaryotes, and I suggest that a SUF-like ancient Fe/S cluster machinery could have been present in the proto-eukaryotic cell or LECA. Current omics data do not provide an answer to this question, but existing efforts to broadly sample the large diversity of archaeal and eukaryotic lineages could provide the missing pieces of this unsolved puzzle.

## Author Contributions

The author confirms being the sole contributor of this work and has approved it for publication.

### Conflict of Interest

The author declares that the research was conducted in the absence of any commercial or financial relationships that could be construed as a potential conflict of interest.
